# Theory-guided Therapeutic Function of Music to facilitate emotion regulation development in preschool-aged children

**DOI:** 10.3389/fnhum.2015.00572

**Published:** 2015-10-14

**Authors:** Kimberly Sena Moore, Deanna Hanson-Abromeit

**Affiliations:** ^1^Department of Music Education and Music Therapy, Frost School of Music, University of MiamiCoral Gables, FL, USA; ^2^Division of Music Education and Music Therapy, School of Music, University of KansasLawrence, KS, USA

**Keywords:** Therapeutic Function of Music, emotion regulation development, preschooler music development, music and arousal, theory

## Abstract

Emotion regulation (ER) is an umbrella term to describe interactive, goal-dependent explicit, and implicit processes that are intended to help an individual manage and shift an emotional experience. The primary window for appropriate ER development occurs during the infant, toddler, and preschool years. Atypical ER development is considered a risk factor for mental health problems and has been implicated as a primary mechanism underlying childhood pathologies. Current treatments are predominantly verbal- and behavioral-based and lack the opportunity to practice in-the-moment management of emotionally charged situations. There is also an absence of caregiver–child interaction in these treatment strategies. Based on behavioral and neural support for music as a therapeutic mechanism, the incorporation of intentional music experiences, facilitated by a music therapist, may be one way to address these limitations. Musical Contour Regulation Facilitation (MCRF) is an interactive therapist-child music-based intervention for ER development practice in preschoolers. The MCRF intervention uses the deliberate contour and temporal structure of a music therapy session to mirror the changing flow of the caregiver–child interaction through the alternation of high arousal and low arousal music experiences. The purpose of this paper is to describe the Therapeutic Function of Music (TFM), a theory-based description of the structural characteristics for a music-based stimulus to musically facilitate developmentally appropriate high arousal and low arousal in-the-moment ER experiences. The TFM analysis is based on a review of the music theory, music neuroscience, and music development literature and provides a preliminary model of the structural characteristics of the music as a core component of the MCRF intervention.

Emotion regulation is an umbrella term to describe interactive, goal-dependent explicit and implicit processes intended to help an individual manage and shift an emotional experience. The unfolding of one’s ability to regulate his or her emotions can be a lifelong process (Ochsner and Gross, 2007), but the primary window of development occurs during the infancy, toddlerhood, and preschool years. In fact, these years provide the critical opportunity for adaptive ER development to occur (Bargh and Williams, 2007; Calkins and Hill, 2007; Eisenberg et al., 2007; Stegge and Terwogt, 2007; Cole et al., 2008; Röll et al., 2012). Atypical ER development is considered a risk factor for mental health problems (Hunter et al., 2011; Röll et al., 2012) and has been implicated as a primary mechanism underlying childhood pathologies (Perry and Pollard, 1998; Zeman et al., 2006; Mullin and Hinshaw, 2007; Thompson and Meyer, 2007; Röll et al., 2012), as well as childhood social competence and school adjustment (Calkins and Hill, 2007; Eisenberg et al., 2007; Jahromi et al., 2012). Furthermore, due to the use-dependent nature of neurodevelopment, structural and functional neural changes associated with atypical ER development affect the functionality of an individual’s brain through adulthood (Perry et al., 1995).

Given the significance of healthy and adaptive ER development for an individual’s mental health, it is important to explore strategies for facilitating its development should an individual be at-risk for maladaptive ER skills. A therapeutic music-based approach may be one way to promote healthy ER development in young children for three reasons:

(1) Music is developmentally appropriate (McDonald and Simons, 1989; Marsh and Young, 2006; Trehub, 2006; Lamont, 2008);
(2) There is a well-documented connection between music, emotions, and physiologic arousal (Meyer, 1956; Berlyne, 1971; James, 1884; Blood and Zatorre, 2001; Menon and Levitin, 2005), even in infants (Trainor and Schmidt, 2003; Trehub, 2003; Parncutt, 2006), whose ER needs are primarily centered on controlling arousal levels (Calkins and Hill, 2007); and
(3) Music is typically used as a way to facilitate ER development through caregiver-child musical interactions (Cross, 2003; Trainor and Schmidt, 2003; Trehub, 2003; Marsh and Young, 2006).

Recent music therapy literature calls for clear and detailed descriptions of music-based interventions to improve the description, procedures, and rationale for music selections (Robb et al., 2011). The TFM intentionally identifies the theoretical framework to support the purpose, description and appropriateness of the musical elements in relation to intended treatment goal and targeted population (Hanson-Abromeit, 2015). Thus, the purpose of this paper is to outline the TFM Plan as a framework for constructing a music-based stimulus to target ER development with preschool-aged children. This TFM Plan provides a set of guidelines grounded in typical developmental competence to support the music therapist’s structure of the music-based stimulus to foster ER development in preschoolers. These guidelines create a basis for clearer intervention reporting and an explicit theory-based rationale of how to structure the music stimulus to more intentionally facilitate therapeutic change. Moreover, the TFM creates greater clarity for the role of the music within an intervention strategy (Hanson-Abromeit, 2015), a core component of the MCRF intervention.

## Emotion Regulation Defined

Emotion is a valenced response to an internal (i.e., perceived or recalled) or external event, situation, or object (Ochsner and Gross, 2005; Gross and Thompson, 2007; Juslin and Sloboda, 2010; Damasio and Carvalho, 2013) that is deemed significant to the individual (Frijda, 2007; Gross and Thompson, 2007). Simplistically, the emotion process is instigated by a situation, which directs an individual’s attention to appraise the situation, then generates a response. This is referred to as the situation-attention-appraisal-response modal model of emotion processing (Gross and Thompson, 2007). Theoretically, ER strategies are embedded in this process (Stegge and Terwogt, 2007) and can influence emotion processing at any point in this sequence (e.g., the situation, attention, appraisal, or response periods) (Gross and Thompson, 2007) through both feedback and feedforward mechanisms (Stegge and Terwogt, 2007). ER is a core characteristic of the emotion process as emotional states typically involve an ER component (Juslin and Sloboda, 2010; Lewis et al., 2010), and it functions in general self regulation as well. Self regulation is the mechanism through which the brain attempts to maximize an individual’s functioning by minimizing distractions (Lewis et al., 2010), with a purpose towards maintaining a sense of equilibrium and homeostasis. Regulatory efforts can occur across multiple domains and it is likely that there is overlap and continuity between them (Calkins and Hill, 2007; Gross and Thompson, 2007). Indeed, it may be difficult to separate ER from other forms of regulation, such as attention regulation and behavioral regulation. With ER, the emotion serves as a signal that equilibrium is disrupted (Bargh and Williams, 2007) and emotion-based regulatory efforts are intended to return an individual back to a state of homeostasis.

A single agreed-upon definition for ER does not yet exist in the literature, but authors tend to agree on several key features. ER is an umbrella term for a diverse set of processes and strategies (Beer and Lombardo, 2007; Calkins and Hill, 2007; Eisenberg et al., 2007; Mullin and Hinshaw, 2007; Gross and Thompson, 2007; Thompson and Meyer, 2007; Lewis et al., 2010; Gyurak et al., 2011). These processes and strategies can be explicit, voluntary, controlled, and conscious (i.e., they are “top–down” strategies) or implicit, reactive, automatic, and subconscious (i.e., they are “bottom–up” strategies) (Beer and Lombardo, 2007; Calkins and Hill, 2007; Gross and Thompson, 2007; Mullin and Hinshaw, 2007; Thompson and Meyer, 2007; Lewis et al., 2010; Gyurak et al., 2011). In reality, this is not an either-or situation as strategies can occur on a continuum from top–down to bottom–up (Gross and Thompson, 2007). The purpose of ER is to manage and shift emotions through dampening, intensifying, or maintaining the intensity and temporal qualities of the emotion experience (Beer and Lombardo, 2007; Calkins and Hill, 2007; Eisenberg et al., 2007; Gross and Thompson, 2007; Thompson and Meyer, 2007). Thus, ER will alter the intensity of the emotion, but not the quality of the emotion itself (Thompson and Meyer, 2007). As a goal-dependent process, how emotions are regulated will depend on an individual’s goal in the given context (Cummings and Davies, 1996; Beer and Lombardo, 2007; Eisenberg et al., 2007; Gross and Thompson, 2007; Thompson and Meyer, 2007). The end result of appropriate ER, therefore, cannot be considered optimal or maladaptive (Thompson and Meyer, 2007) as it should be congruent with what an individual needs in that particular moment or situation. Finally, ER is a dynamic and interactive process (Cummings and Davies, 1996; Calkins and Hill, 2007; Gross and Thompson, 2007; Gyurak et al., 2011) that does not rely solely on modifying emotions, but also on the individual continuing to monitor and appraise the emotions (Thompson and Meyer, 2007). Considering these key characteristics, for the purposes of this paper ER will be defined as a concept that describes interactive, goal-dependent explicit and implicit processes intended to help an individual manage and shift an emotional experience.

## Emotion Regulation Development

The unfolding of one’s ability to regulate his or her emotions can be a lifelong process (Ochsner and Gross, 2007), but the primary window of development occurs during the infant, toddlerhood, and preschool years. Although there are multiple possible pathways to ER development, the general trajectory follows a three-stage arc: (1) simple physiologic and reflexive responses (Calkins and Hill, 2007), (2) caregiver-directed co-regulatory strategies or the use of simple attentional and motor strategies (Eisenberg et al., 2007; Thompson and Meyer, 2007), and (3) active and intentional self-regulation of emotions (Zeman et al., 2006; Bargh and Williams, 2007; Calkins and Hill, 2007; Eisenberg et al., 2007; Stegge and Terwogt, 2007; Thompson and Meyer, 2007; Cole et al., 2008). Although an innate developmental template for ER development exists, ER skills are also socially constructed (Thompson and Meyer, 2007). These socially constructed skills are influenced by one’s cultural experiences, family environments, caregiver interactions, and gender expectations.

In the 1st year of life, ER needs are primarily centered on controlling arousal levels (Calkins and Hill, 2007), managing emotional cues, and handling external and internal stress (Feldman, 2009). They tend to involve either innate physiological mechanisms, such as a generalized approach or withdrawal response to an arousal-inducing stimulus (Calkins and Hill, 2007), or passive, caregiver-directed, mutual regulation (Thompson and Meyer, 2007; Feldman, 2009). Caregiver interactions strongly mediate the development of ER. This is an important consideration as children generally cope more adaptively and develop more appropriate ER strategies when caregivers respond supportively and sympathetically to their emotional expressions (Perry and Pollard, 1998; Gross and Thompson, 2007; Thompson and Meyer, 2007; Koole, 2009). During infancy, sensitive, flexible, and responsive caregiving behaviors become integrated into an infant’s repertoire of ER responses (Calkins and Hill, 2007). Such behaviors influence the infant’s ER development by demonstrating that stress can be managed (Thompson and Meyer, 2007; Cole et al., 2008) and that an adult can help with the management (Thompson and Meyer, 2007). Furthermore, it is not just through calming a distressed infant that caregivers support ER development but also through stimulating the infant by engaging in face-to-face play, an activity that emerges around 2–3 months of age (Thompson and Meyer, 2007). These calming and stimulating interactions offer multiple and frequent opportunities to practice emotion and arousal regulation (Zeman et al., 2006). They strongly influence developing ER capacities in the infant and support continued ER development during the toddler and preschool years.

During the toddler years, caregivers facilitate the shift from passive co-regulation to active self-regulation of emotions by incorporating a variety of strategies and directives designed to facilitate, prompt, model, and structure the emotion experience (Zeman et al., 2006; Calkins and Hill, 2007; Thompson and Meyer, 2007). Caregiver strategies may include preemptively structuring the environment to help control for emotional demands, providing social referencing cues, prompting the use of specific ER strategies, or providing contingencies for behaviors (Zeman et al., 2006; Thompson and Meyer, 2007). Caregiver directives are also important as they facilitate and externally “coach” the transition from passive co-regulation of emotions to more active, internal self-regulation of emotions. Caregivers utilize a variety of directives, which may include distracting the toddler, helping the toddler problem-solve, providing alternate interpretations of situations, providing social referencing cues, suggesting adaptive responses, offering alternatives for maladaptive behavior, or structuring experiences to help make emotional demands more manageable (Thompson and Meyer, 2007). In addition to the caregiver-facilitated shift toward more active self-regulation of emotions, ER development toward the end of toddlerhood is characterized by an emerging use of objects (e.g., toys), social interactions (Eisenberg et al., 2007), and cognitive strategies (Stegge and Terwogt, 2007) to facilitate ER.

Emotion regulation development in the preschool years, defined here as ages three through five years, is generally characterized by a decline in caregiver interventions and directives (Thompson and Meyer, 2007), a greater emphasis on top–down cognitive strategies (Stegge and Terwogt, 2007), a growing repertoire of behavioral ER strategies, and an increasing understanding and use of culturally defined behavioral display rules (i.e., cultural and commonly gender-based expectations for how an individual shows emotions in a given situation) (Zeman et al., 2006). The caregiver–child relationship continues to be of great importance during the preschool years though there is a shift in how that relationship informs ER development. The “coaching” type of ER development that began during the toddlerhood years persists during the preschool years as caregivers continue to facilitate the transition to active self-regulation of emotions. Caregivers still use a variety of strategies and directives designed to facilitate, prompt, model, and structure the emotional experience (Zeman et al., 2006; Calkins and Hill, 2007; Thompson and Meyer, 2007). These may include preemptively structuring the environment to help control for emotional demands, providing social referencing cues, prompting the use of specific ER strategies, and providing contingencies for behaviors (Zeman et al., 2006; Thompson and Meyer, 2007). However, the employment of these strategies and directives decline during this developmental period as preschoolers take a more active role in the regulation of their emotions (Thompson and Meyer, 2007).

A primary goal during the preschool developmental period is for children to begin to identify appropriate and inappropriate ER strategies and to assess the effectiveness of these strategies (Cole et al., 2008; Röll et al., 2012). Furthermore, unlike earlier years where children are unable to select or modify their environments and situations (Gross and Thompson, 2007), preschool-aged children have a greater ability to utilize strategies designed to alter a situation (Cole et al., 2008). Around age three years, children begin to develop successful inhibitory control (Bargh and Williams, 2007) and the next two years are characterized by an emerging explicit awareness of ER strategies (Cole et al., 2008). Caregiver-preschooler conversations about emotions facilitate ER development as they convey cultural values, gender expectations (Zeman et al., 2006; Thompson and Meyer, 2007), and assist the child in identifying emotions and regulating negative affect (Zeman et al., 2006).

### Childhood Stress and ER Development

Emotions can be viewed as homeostasis-disrupting events as they indicate an organism is not in a steady and calm state of equilibrium (Bargh and Williams, 2007). Furthermore, ER development in infancy centers on controlling arousal levels (Calkins and Hill, 2007) and handling internal and external stress (Feldman, 2009). In a mature individual, the classic “fight, flight, or freeze” stress response is the body’s adaptive response to a stressful event, such as occurs during an emotion-inducing event (Perry and Pollard, 1998). In this instance, although the emotion offers an important clue about one’s environment, it can also be considered a homeostasis-disrupting event (Bargh and Williams, 2007). Thus, one primary function of ER is to return the organism to a state of homeostasis through managing and shifting the physiological response associated with the emotion experience. When these efforts are not successful, an individual remains in a stressed, disequilibrium state and is said to be dysregulated (Linehan et al., 2007). Given these connections, linking ER development to the childhood stress response is essential.

Stress responses exhibit differently in children than in adults. Instead of the classic stress response, a child’s stress response generally takes one of two patterns, (1) hyperarousal, also referred to as hyperactivating or overregulation, or (2) dissociative, also referred to as deactivating or underregulation (Perry et al., 1995; Cummings and Davies, 1996; Perry and Pollard, 1998; Mikulincer et al., 2003; Mullin and Hinshaw, 2007). The type of response pattern a child utilizes is formed in infancy and influenced by caregiver–infant interactions. When an infant experiences stress, its initial reaction is hyperarousal (e.g., crying) as it seeks proximity to the caregiver (Perry et al., 1995; Perry and Pollard, 1998). If this strategy works, the infant will continue to use this strategy when he or she seeks proximity, love, and support (Mikulincer et al., 2003). If the initial hyperarousal response attempt does not work, the infant will disengage from its proximity-seeking behavior and will instead attempt to manage the stress without caregiver support. These self-soothing and “managing” behaviors are on the dissociative end of the continuum and can manifest in behaviors such as distraction, avoidance, numbing, daydreaming, and fainting (Perry et al., 1995; Perry and Pollard, 1998; Mikulincer et al., 2003). The type of stress response a child utilizes is also mediated by age and gender as younger children and females are more likely to use dissociative strategies (Perry et al., 1995).

## Developmental Implications

Emotion regulation development occurs in infancy and early childhood and is heavily influenced by the caregiver–infant relationship (Schore, 2001; Calkins and Hill, 2007; Eisenberg et al., 2007; Thompson and Meyer, 2007). Due to the use-dependent nature of neurodevelopment (Perry and Pollard, 1998), early stress-inducing emotional experiences shape the structure and function of the developing brain. Thus, without being exposed to developmentally appropriate ER experiences, a child is at-risk for developing poor ER skills and maladaptive ER strategies. This has implications for an individual’s behavioral response patterns (Perry et al., 1995; Perry and Pollard, 1998; Calkins and Hill, 2007; Eisenberg et al., 2007; Mullin and Hinshaw, 2007; Feldman, 2009; Lewis et al., 2010; Jahromi et al., 2012), emotional and social health (Perry et al., 1995; Zeman et al., 2006; Calkins and Hill, 2007; Eisenberg et al., 2007; Blair et al., 2008; Feldman, 2009; Lewis et al., 2010; Jahromi et al., 2012), cognitive skills and learning (Perry et al., 1995; Schore, 2001; Calkins and Hill, 2007; Blair et al., 2008; Jahromi et al., 2012), and the potential development of psychopathology (Perry and Pollard, 1998; Zeman et al., 2006; Mullin and Hinshaw, 2007; Thompson and Meyer, 2007; Hunter et al., 2011; Röll et al., 2012). In short, the development of adaptive ER skills affects a child’s mental health, behavioral and emotional responses to stress, his or her ability to develop healthy and appropriate adult-child and peer relationships, and the child’s ability to learn in school.

Children are at-risk to develop maladaptive ER skills regardless of whether they exhibit hyperarousal or dissociative stress response patterns. For example, a link exists between hyperarousal response patterns and uncontrolled “acting out” or aggressive behaviors. Increased amounts of stress in infancy and childhood may alter the developing HPA axis, which could result in a dysregulated stress response system (Perry and Pollard, 1998; Calkins and Hill, 2007). The effect of increased stress that occurs prenatally and during infancy can have even more damaging and lasting effects, since one of the first systems to mature are basic physiological systems implicated in the stress response. These physiological systems are integrated into, and mediate the development of, later-developing emotional, cognitive, and behavioral systems (Calkins and Hill, 2007). A dysregulated stress response system may lead to heightened states of arousal, which is commonly found in aggressively reactive children (Mullin and Hinshaw, 2007). This type of aggressive reactivity can be accompanied by difficulty in controlling such reactivity, as these children may exhibit low levels of top–down, cognitive-based ER strategies (Eisenberg et al., 2007). In addition to increased reactivity, children who have hyperarousal response patterns may exhibit other types of externalizing behaviors, such as inattention, impulsivity, anxiety, hyperactivity, hypervigilance, and antisocial behaviors (Perry and Pollard, 1998; Mullin and Hinshaw, 2007). Such patterns places these children at-risk for externalizing disorders (Zeman et al., 2006) and childhood pathologies such as Attention-Deficit/Hyperactivity Disorder (ADHD), Oppositional Defiant Disorder (ODD), and Conduct Disorder (CD) (Mullin and Hinshaw, 2007).

A child is also at-risk when dysregulation occurs at the dissociated end of the childhood stress response continuum. In these instances, maladaptive ER skills are characterized by an inhibition or overcontrol of an emotion (Calkins and Hill, 2007). Children who exhibit this response pattern are thought to be as highly reactive as their hyperaroused counterparts, while their behavioral responses are characterized by poor attention regulation, poor behavior initiation (Eisenberg et al., 2007), and maladaptive self-soothing behaviors such as rocking or “cutting” (Perry and Pollard, 1998). In addition, they are prone to internalizing problems and disorders (Zeman et al., 2006; Eisenberg et al., 2007; Röll et al., 2012). Having said that, the link between maladaptive ER skills and internalizing problems is not as clear as it is between maladaptive ER skills and externalizing problems (Eisenberg et al., 2007).

Overall, ER significantly influences an individual’s ability to function, which makes the development of ER an important area to understand. These effects have also been noted beyond childhood such that adaptive ER may be a marker of poor mental health (Cole et al., 2008; Gyurak et al., 2011; McRae et al., 2012). How stress and adversity is experienced and handled early in life may program an adult’s stress response (Hunter et al., 2011). This does not mean that every child who experiences stress is susceptible to such challenges and problems; ER development outcomes also depend on the child’s natural temperament and resilience (Ochsner and Gross, 2007) and the environmental context (Mullin and Hinshaw, 2007).

A child’s maladaptive ER-related behaviors serve an important communicative function for parents, caregivers, and clinicians as they indicate that the child is in a dysregulated state. Chronically maladaptive behaviors may suggest a pervasive problem that needs to be addressed. The hyperarousal or dissociative behaviors themselves, although observable, are not the primary issues of concern when facilitating ER development. The underlying problem concerns the development of maladaptive ER skills. Therefore, understanding how ER develops is key to effective intervention methods and supports the call for a more theory-based approach to clinical work, including disciplines such as music therapy (Robb, 2012). A theory-based approach should focus less on the hyperarousal and dissociative behaviors and more on the mechanisms underlying ER development—the developing stress response system, the supportive, caring environments, the predictable, safe, flexible, and loving caregiver responses, and the interactions between them. These are the mechanisms that influence brain development and should theoretically be the primary targets of interventions intended to facilitate ER development.

## Music as an ER-Facilitating Mechanism

Numerous therapeutic techniques and training programs exist to help improve ER in preschoolers. However, a need still exists for approaches that incorporate a wider range of bottom–up and top–down strategies, provide in-the-moment opportunities to manage “stress” (e.g., emotionally arousing experiences), and afford opportunities for this practice to be realized in the context of a developmentally appropriate interactive adult-child relationship. One such therapeutic approach that may fit these needs is a music-based intervention.

The idea that music can induce emotions began to emerge in the scientific literature in the late 1800s (James, 1884) and continued to be mentioned and explored in subsequent literature from the psychological and anthropological fields (James, 1884; Meyer, 1956; Merriam, 1964; Sears, 1968; Berlyne, 1971; Lazarus, 1991; Zajonc, 1994; Frijda, 2007). The connection between music and emotions was largely ignored in the latter half of the 20th century; however, there has been renewed interest over the past 20 years in understanding this phenomenon (Juslin and Sloboda, 2010). It is generally agreed that music evokes emotions and stimulates physiological and behavioral responses (Habibi and Damasio, 2014). In addition, more recent neuroscience research indicates there are shared neural networks implicated in both emotion and music processing (Blood and Zatorre, 2001; Satoh et al., 2001; Brown et al., 2004; Menon and Levitin, 2005; Baumgartner et al., 2006; Koelsch et al., 2006, 2008; Bengtsson et al., 2007; Brown and Martinez, 2007; Foss et al., 2007; Kleber et al., 2007; Mitterschiffthaler et al., 2007; Mizuno and Sugishita, 2007; Berkowitz and Ansari, 2008; Limb and Braun, 2008; Lerner et al., 2009), and specifically between music and ER processing (Sena Moore, 2013). There is also evidence to support the developmentally appropriate use of music-based experiences to target ER development in preschoolers because music stimulates physiologic arousal and induces emotions, and assumes a natural role in bonding and social interactions.

### Developmental Appropriateness

Parents and professionals who work with preschool-aged children know that they are inherently musical. This connection is also well documented in the literature. Preschoolers have an unbridled enthusiasm for music (Trehub, 2006) and music has a prevalent role in their lives (Lamont, 2008). The developmental foundations for music are laid in infancy. Infants are born with a curiosity and attentiveness to musical sounds (McDonald and Simons, 1989; Tafuri, 2008), and with finer perception of frequency, timing, and timbre than what is needed at this point in their musical development (Trehub, 2003). As preschoolers, a child’s first social experiences likely involve musical games (McDonald and Simons, 1989; Marsh and Young, 2006). Furthermore, music holds an important role in childcare rituals and routines, both in the home and preschool or daycare settings (Lamont, 2008). Thus, it seems natural and acceptable to utilize a medium with which preschool children are familiar and are inherently drawn to as a therapeutic mechanism.

### The Music – Physiologic Arousal – Emotions Connection

The connection between music, physiologic arousal, and emotions has been recorded as early as the late nineteenth century (James, 1884). More recently, neural structures involved in emotion processing, including the striatum, amygdala, ventromedial prefrontal, anterior cingulate cortex (Damasio, 2000), and insula (Damasio and Carvalho, 2013), have also been implicated in music processing (Habibi and Damasio, 2014). Furthermore, listening to music produces physiologic changes associated with emotion processing. What is particularly notable is that an overt reaction is not typically required for musically induced emotions (Trainor and Schmidt, 2003).

Another notable observation is how early this connection begins. Infants have demonstrated sensitivity to sound and movement patterns and their emotional connotations (Parncutt, 2006). Caregiver–infant interaction patterns mirror one another using music-like qualities that engage infant attention, communicate affective responses, encourage social reciprocity, and facilitate language (Trevarthen and Aitken, 2001). Thus, there is a connection between music and physiologic arousal as a means of modulating an infant’s arousal level. This can be seen through caregiver–infant interactions, which often incorporate stimulating music (i.e., play-songs) and calming music (i.e., lullabies) (Trainor and Schmidt, 2003; Trehub, 2003). In addition, music has the ability to convey emotional information (Schubert and McPherson, 2006), even for young children (Trainor and Schmidt, 2003). Preschoolers are able to distinguish basic emotions, such as happy, sad, anger, and fear, and express them through music (Stachó et al., 2013). Thus, the connection between music, physiologic arousal, and emotion induction apparent early in the development process provides additional support for the use of music as a mechanism to facilitate ER development in preschool children.

### Music-mediated Bonding and Interactions

Perhaps the strongest argument supporting the use of music to facilitate ER development may be its role in facilitating bonding and caregiver–child interactions. From an early age children are exposed to musical interactions, first with their caregivers (Cross, 2003; Trainor and Schmidt, 2003; Trehub, 2003; Barrett, 2006; Custodero, 2006; Marsh and Young, 2006) and then through social experiences (McDonald and Simons, 1989; Marsh and Young, 2006) and interactions in a daycare or preschool setting (Lamont, 2008). Caregivers commonly use music as part of their familiar and structured parenting rituals (Barrett, 2006, 2011; Custodero, 2006; Lamont, 2008). These early music interactions help to stimulate the child, soothe the child, or allow the child to share emotional information (Trehub, 2009). Furthermore, caregiver–infant interactions seem to incorporate music-like characteristics in that they have a rhythmic and dynamic back-and-forth quality. Such music-like interactions are critically important in helping a child acquire capacities to self-regulate and to bond emotionally with another person (Cross, 2003) and they serve an essential role in nonverbal caregiver-infant communications (Trevarthen and Aitken, 2001; Marsh and Young, 2006) that begin forming before birth (Welch, 2006b; Tafuri, 2008). Grounded in these early experiences, spontaneous singing emerges during early childhood frequently functioning as way to express and regulate the young child’s emotions (Barrett, 2006, 2011).

Even beyond infancy and the caregiver–infant relationship, a key characteristic of musical play is its importance as a form of social interaction (Marsh and Young, 2006). Interactive musical play between parents and young children can have a positive effect on the quality of parent–child communication and understanding (Welch, 2006b). Outside the home, music infuses the interactions between young children and their peers. They share musical play ideas, synchronize rhythmic movements with each other, and imitate each other’s melodic ideas (Marsh and Young, 2006). As a natural component of early interactions, bonding, and social experiences, music can be an effective mechanism to facilitate therapeutic change in preschool children.

### Neural Support

There is emerging, although inconclusive, neurological support for using music as an intervention mechanism that centers on the role of the right hemisphere in music processing, attachment, and stress. Infants show a right hemisphere advantage for music processing (Trehub, 2003) and it is the right hemisphere that seems specialized for processing musically induced emotions (Peretz, 2010). In addition, the prosodic elements of speech processing, the patterns of stress and intonation that are sometimes referred to as the “music” of speech, may be processed more in the right hemisphere than in the left (Welch, 2006b). Outside of music and speech processing, the right hemisphere is implicated in an organism’s ability to cope with stress (Schore and Schore, 2008). Furthermore, caregiver–infant interactions are implicated in the appropriate development of the prefrontal cortex, particularly in the right hemisphere (Calkins and Hill, 2007). Finally, although this does not address ER from a developmental perspective, music activates neural networks implicated in ER processing (Sena Moore, 2013). This means that, although a specific brain-based connection has not yet been made, evidence for a connection between music processing, attachment, stress management, and music and ER exists. Such correlations provide support for pursuing a line of inquiry that explores how music can be used to facilitate ER development.

## Therapeutic Functions of Music for Targeting ER Development

Strong evidence exists for the use of a music-based treatment approach to facilitate ER development. An important aspect of an effective music-based intervention is to determine how to structure the music stimulus intentionally for this task. One method to develop effective music-based interventions is to design a TFM Plan for the proposed strategy (Hanson-Abromeit, 2015). The TFM has been defined as “the direct relationship between the treatment goal and the explicit characteristics of the musical elements, informed by a theoretical framework and/or philosophical paradigm in the context of a client” (Hanson-Abromeit, 2013). In other words, it allows the clinician to have an explicit understanding of why and how music affects a desired change, thus informing the intentional, therapeutic use of music in clinical practice. For the purposes of this paper, the goal in outlining the TFM Plan is to help determine how to structure a music stimulus so that it is developmentally appropriate for preschool-aged children and can have either a stimulating (“high arousal”) or a calming (“low arousal”) effect, core components of the music-based MCRF intervention. This analysis is based on a review of music theory, music neuroscience, and music development literature resulting in a descriptive synthesis of the musical characteristics reflective of high and low arousal musical stimuli appropriate for preschool-aged children. The TFM Plan (Hanson-Abromeit, 2015) is detailed in Supplementary Table 1.

### Synthesis of Developmentally Appropriate Music for Preschool-aged Children

Developmentally appropriate music stimuli should be predictable and structured, incorporating rhythmic and melodic repetition and simple consonant harmonies. Melodies should have an easy-to-follow contour characterized by descending intervals and step-wise movements. Pitches and pitch intervals should help create “singable melodies” by falling within an octave pitch range and include skips with small-integer ratio intervals (e.g., octaves, perfect fifths, perfect fourths). The music should mostly incorporate binary rhythms, should have a simple form, and should be primarily diatonic. Stylistically appropriate music should sound like popular music, be chant-like and repetitive, or be solitary and free-flowing. Preschool-aged children can be expected to synchronize their motor movements to a beat, detect tempo changes and synchronize to them, and control and alter basic rhythmic patterns. Most will have an imprecise sense of pitch, but will posses a developing ability to sing in tune and can produce and discriminate loud and soft sounds. Furthermore, they should be able to discriminate various musical styles, timbres, and textural changes. If incorporating valence into the music-based experience (e.g., a musically induced positive or negative emotion), major modes can be used to reflect positive emotions and minor modes negative ones. Preschoolers can be expected to focus on words, thus lyrics provide a verbal prompt to use an explicit ER strategy or explore the effectiveness of an ER strategy. This synthesis is based on the following literature: Krumhansl and Keil, 1982; Trehub et al., 1986; Drake and Gérard, 1989; McDonald and Simons, 1989; Morrongiello and Roes, 1990; Schwarzer, 1997; Drake et al., 2000; Dalla Bella et al., 2001; Costa-Giomi, 2003; Drake and Bertrand, 2003; Trainor and Schmidt, 2003; Trehub, 2003, 2006, 2009; Peretz and Zatorre, 2005; Devous et al., 2006; Leighton and Lamont, 2006; Marsh and Young, 2006; Schubert and McPherson, 2006; Welch, 2006a; Marshall and Hargreaves, 2007; Miyamoto, 2007; Lamont, 2009; Patel, 2009; Stewart et al., 2009; Trainor and Zatorre, 2009; Gabrielsson and Lindström, 2010; Tsang et al., 2011.

### Synthesis of High Arousal Music

A variety of characteristics can be combined to create highly arousing music, and a music stimulus can be manipulated in various ways to make it more arousing. Highly arousing music avoids ritardandos and accents on unstable (i.e., rhythmically unstressed) notes. For preschool children, it may include complex ternary rhythmic patterns. High arousal music can also have rising pitches and use instruments that produce extraneous harmonic “noise.” It typically will be in a fast tempo with bright or sharp timbres, rising or sharp micro-intonations, fast or shallow vibrato, staccato articulations, quick and abrupt attacks, complex musical textures, or with variable articulation styles. Lyrics can be created to reflect the intended arousal level.

To manipulate a music stimulus to make it more arousing, sudden and unexpected musical events or novel musical elements can be incorporated. These can be melodic (e.g., ascending intervals, wide skips, and intentional mis-tunings), pitch-related (e.g., sharp change in pitch tuning), timbral (e.g., novel timbres or unexpected timbral changes), stylistic (e.g., sudden change in musical style), rhythmic (e.g., sudden or sharp rhythmic changes), or textural (e.g., novel texture or unexpected textural changes). If incorporating valence into the music experience, it may help to reference a two-dimensional circumplex model that features the dimensions of valence and activity, or arousal. Within this model, happiness, anger, and fear are considered emotions of high arousal (Juslin and Timmers, 2010). Happy-sounding music can be loud, have small tempo variations, and variability in loudness levels. Angry music can be loud and have small tempo variations. Fearful-sounding music can be soft and have substantial variability in terms of volume or tempo. Finally, creating a sense of expectation is an important element of a music-induced emotional response (Stevens and Byron, 2009) and another mechanism through which arousal can be elicited. Musically, expectation can occur by creating a pause in the melodic or rhythmic pattern or through the use of harmonic dissonance. This synthesis is based on the following literature: Trehub et al., 1986; Morrongiello and Roes, 1990; Dalla Bella et al., 2001; Costa-Giomi, 2003; Drake and Bertrand, 2003; Trainor and Schmidt, 2003; Peretz and Zatorre, 2005; Devous et al., 2006; Schubert and McPherson, 2006; Lamont, 2009; Patel, 2009; Stevens and Byron, 2009; Stewart et al., 2009; Trainor and Zatorre, 2009; Gabrielsson and Lindström, 2010; Juslin and Timmers, 2010; Mote, 2011; Yrtti, 2011; Huron, 2013.

### Synthesis of Low Arousal Music

Structuring music to create a calming, low arousal effect will incorporating the familiar developmentally appropriate musical characteristics previously outlined. Other music characteristics that may be considered low arousal for preschoolers include music in a lower-than-normal range, no changes in pitch tuning, soft loudness levels, narrow loudness variability, familiar, soft, or dull timbres, slow tempos, legato articulations, slow attacks, simpler textures, slow vibrato, and limited articulation variability. Rhythmic characteristics of low arousal music include incorporating a ritardando at the end of a song, placing accents on stable (i.e., rhythmically stressed) notes, and avoiding rhythmic change. Additionally, lyrics can be created to reflect the intended arousal level. This synthesis is based on the following literature: Trainor and Schmidt, 2003; Peretz and Zatorre, 2005; Devous et al., 2006; Schubert and McPherson, 2006; Patel, 2009; Stewart et al., 2009; Trainor and Zatorre, 2009; Gabrielsson and Lindström, 2010; Juslin and Timmers, 2010; Mote, 2011; Yrtti, 2011; Huron, 2013.

## TFM Application to Intervention: Musical Contour Regulation Facilitation

One of the primary goals of ER development during the preschool years is to practice different ER strategies in various situations and contexts. It is through practicing these strategies that ER development transitions from being explicit, top–down, caregiver-facilitated processes to implicit, automatic, self-initiated ones (Bargh and Williams, 2007). Furthermore, typical ER development is mediated in large part through the child’s interactive experiences with a caregiver (Calkins and Hill, 2007; Eisenberg et al., 2007; Thompson and Meyer, 2007). A child may be at-risk for developing maladaptive ER strategies if he or she does not have a secure attachment relationship with a primary caregiver and is not exposed to experiences that help him or her practice ER strategies in an adaptive and safe way (Calkins and Hill, 2007).

Preschool-aged therapeutic strategies explicitly designed to facilitate the practice of ER strategies within the context of a trusted relationship remains extremely limited, with one notable exception (Betty, 2013). Many of the published therapeutic strategies are verbal- or behavioral-based (Webster-Stratton and Reid, 2003; Izard et al., 2008; Johnson, 2012) and although they incorporate evidence to support the efficacy of the particular treatment technique or training program, several limitations exist. First, such strategies only target verbal, top–down ER strategies, even though ER strategies can occur on a continuum from top–down to bottom–up (Gross and Thompson, 2007). Second, there is a separation between the timing of the treatment approaches and the occurrence of an emotionally stressful situation. Children cope more adaptively and acquire more positive ER strategies when caregivers respond supportively and sympathetically to their emotional expressions as they occur (Perry and Pollard, 1998; Gross and Thompson, 2007; Thompson and Meyer, 2007; Koole, 2009). In other words, current approaches are a priori therapeutic methods that may help the child learn ER strategies, but may not provide the child with an opportunity for real-time management of emotionally arousing experiences necessary to internalize the strategies. Third, ER is largely learned through caregiver–child interactions. An a priori therapeutic approach may help the child learn ER strategies, but it does not mean that the child will get the interactions needed to practice and internalize the strategies. Although these limitations are partially addressed through the parent training component included in some programs (i.e., parents are trained to provide necessary in-the-moment responsiveness to an emotionally stressed child) (Webster-Stratton and Reid, 2003; Izard et al., 2008), what may be needed is a therapeutic approach that incorporates real-time, in-the-moment opportunities to practice experiencing and managing stressful experiences.

Music may function as a mechanism to provide in-the-moment, interactive opportunities for the management and regulation of “stress” (e.g., emotionally arousing experiences) in the context of a healthy adult–child relationship. Behavioral and neural evidence supports using music as the mechanism due to music’s natural role in infant and early childhood interactions (McDonald and Simons, 1989; Cross, 2003; Trainor and Schmidt, 2003; Trehub, 2003; Marsh and Young, 2006; Welch, 2006b; Lamont, 2008), caregiver-infant bonding (Cross, 2003; Marsh and Young, 2006), and developmental appropriateness (McDonald and Simons, 1989; Trehub, 2003, 2006; Marsh and Young, 2006; Lamont, 2008). Based on this support, the synthesized TFM Plan identifies the structural characteristics of the musical elements to compose preschool-aged developmentally appropriate (neutral arousal), arousing (high arousal), and calming (low arousal) musical experiences (see Supplementary Table 1). This TFM Plan provides the theory-based rationale for music stimuli purposefully constructed for an intervention context.

The MCRF intervention was designed with the intention that the contour and temporal structure of a music therapy session alternate between high- and low-arousal states in a way that theoretically mirrors the changing flow of the caregiver–infant interaction. In essence, the MCRF intervention does not seek to induce or elicit specific emotions, but to use the music stimulus to manipulate the arousal levels in preschool-aged children, exposing them to alternation of stimulating and calming experiences (Sena Moore, 2014, 2015). This strategy builds on the works of Meyer (1956), Berlyne (1971), and others (Juslin and Sloboda, 2010), as it is intended to manipulate the physiologic aspect of the emotion process, (Zelazo and Cunningham, 2007; Koole, 2009; Juslin and Sloboda, 2010), through music-based experiences. Music stimuli for the MCRF intervention was composed based on guidelines that emerged from the TFM Plan (Hanson-Abromeit, 2015). An intervention manual was designed to provide a clear and detailed description of the MCRF intervention protocol (Sena Moore, 2014). Manualization of interventions improves intervention transparency and subsequent transfer of music-based interventions from research to clinical practice, thus the information included in this manual adheres to music-specific intervention reporting guidelines (Robb et al., 2011).

It is expected that exposing preschool-aged children to alternating characteristics of the musical elements will provide opportunities for them to practice managing high and low arousal experiences in the moment. Furthermore, it is expected that over time, this practice will lead to improvements in ER skills and in their ability to manage high and low arousal situations as measured by changes in hyperarousal and dissociative stress response pattern indicators (i.e., an increased use of adaptive ER skills, increased attending behaviors, decreased emotional reactivity, and decreased aggressive behaviors). As an initial exploration, a study was conducted to provide an examination of the utility of using music as a way to facilitate ER development in preschool-aged children (Sena Moore, 2015). More specifically, as a first step in a phased research agenda, the study explored the feasibility of the MCRF intervention as a way to improve ER abilities in typically developing preschoolers. Utilizing an embedded convergent mixed methods design, the researcher examined whether the MCRF intervention showed promise of being successful, and explored its acceptability and ease of integration as perceived by parents and teachers. Preliminary findings were encouraging. Most parents and teachers noted emotion-based changes in the children following MCRF treatment and they all believed in the importance and helpfulness of music on developmental outcomes. Furthermore, analyses exploring the efficacy of the MCRF intervention indicated clinically significant improvements in ER skills in the children following treatment. Together, these findings endorse future normative and clinical study of the MCRF intervention as way to facilitate ER development, especially as this medium is highly desired by parents and teachers and can be easily integrated in a preschool setting.

## Conclusion

The purpose of this paper is to provide a theoretical rationale for the TFM Plan, with specific application to a music-based ER intervention strategy for preschool-aged children, the MCRF intervention (Sena Moore, 2014, 2015). The primary window for appropriate ER development occurs during the infancy, toddlerhood, and preschool years. Atypical ER development is considered a risk factor for mental health problems and has been implicated as a primary mechanism underlying childhood pathologies, as well as childhood social competence and school adjustment. Furthermore, due to the use-dependent nature of neurodevelopment, structural and functional neural changes associated with atypical ER development affect the functionality of an individual’s brain through adulthood. Current treatment approaches and training programs are primarily verbal- and behavioral-based. Most of them incorporate top–down ER strategies, which represent only one end of the explicit-to-implicit ER strategy continuum. Furthermore, a disconnect occurs between the timing of the treatment and the need to handle emotionally charged situations in the moment, as well as a lack of the caregiver-child interactive component that is central to typical ER development. Limited therapeutic options exist to provide real-time, adult-child interactive opportunities to manage emotionally arousing experiences and simultaneously practice and internalize ER strategies

The incorporation of intentional music experiences may be one way to address these limitations. Behavioral and neural evidence supports the use of music as the mechanism for such experiences due to music’s developmental appropriateness, as well as its natural role in infant and early childhood interactions and caregiver-infant bonding. The MCRF intervention was designed with the intention that the contour and temporal structure of a music therapy session alternate between high- and low-arousal states in a way that theoretically mirrors the changing flow of the caregiver–infant interaction. In essence, this method does not seek to induce or elicit specific emotions, but to use the music stimulus to manipulate the arousal levels in preschool-aged children, exposing them to alternation of stimulating and calming experiences. This paper proposes that the music characteristics can be specifically designed to provide in-the-moment, interactive opportunities for stress management (e.g., of emotionally arousing experiences) and regulation in the context of a healthy adult–child relationship.

Results from the TFM Plan provided preliminary guidelines as to how to structure and manipulate the music stimulus in the MCRF intervention. The TFM described in this paper is a theory-based construct for music stimuli specific to an intervention strategy intended to practice real time ER within a therapist–preschooler relationship. It should be noted that the music stimulus guidelines outlined in this paper only account for Western musical practices. It is outside the scope of this review to consider other cultural music contexts, although the process for analyzing the TFM could transfer to these situations. In addition, there are individual and sociocultural influences to consider when planning how to structure the music stimulus and the TFM Plan should be adapted accordingly for each given situation. For example, the TFM Plan in this paper is appropriate ER experiences for typically developing children aged 3–5. Adaptations may be needed if working with children with a history of complex trauma as they will have unique individual and culturally influenced needs that are not considered in the TFM analysis outlined in this paper.

Further study of the implementation of the MCRF intervention is warranted. Additionally, careful analysis of the music within the context of an intervention strategy will support the reliability and fidelity of the TFM Plan for ER. Greater understanding and reporting of the characteristics of the music-based stimuli as suggested through the TFM Plan (Hanson-Abromeit, 2015) will contribute to the continued refinement of the TFM for preschool-aged ER, the generalizability of the music stimuli to other intervention strategies, and a deeper understanding of the role of music as a mechanism for ER development.

## Author Contributions

KSM contributed to the design, acquisition, analysis, and interpretation of the work, drafted the work, and gave approval of the version to be published and agrees to be accountable for all aspects of the work including accuracy and integrity of the work. DH-A contributed to the conception and interpretation of the work and critical revision for important intellectual content, and gave approval of the version to be published and agrees to be accountable for all aspects of the work including accuracy and integrity of the work.

## Conflict of Interest Statement

The authors declare that the research was conducted in the absence of any commercial or financial relationships that could be construed as a potential conflict of interest.

## ABBREVIATIONS

ER: emotion regulation
MCRF: Musical Contour Regulation Facilitation
TFM: Therapeutic Function of Music
