# Supplementary material for: Theory-guided Therapeutic Function of Music to facilitate emotion regulation development in preschool-aged children
Source: Front Hum Neurosci. 2015 Oct 14;9:572. doi: 10.3389/fnhum.2015.00572 (PMC4604312; doi:10.3389/fnhum.2015.00572)
Supplement: Supplementary file 1 [file Table_1.PDF]

Table 1

Therapeutic Functions of Music Plan (Hanson-Abromeit 2015)

Goal: To provide preschool-aged children musically facilitated opportunities for real-time modulation of high and low arousal experiences.

Musical Element	Theoretical Framework	Purpose of Musical Element	Description of Musical Element
Melody	<u>Music Theory</u><br>• Melodic contour/direction has little effect on emotional expression (Gabrielsson and Lindström 2010).<br>• Melodic structure can be used to create expectation (Stevens and Byron 2009).<br><br><u>Music Development</u><br>• Children possess concepts and control of melodic contour (Costa-Giomi 2003; McDonald and Simons 1989; Morrongiello and Roes, 1990; Schwarzer 1997; Welch 2006).<br>• Songs should contain melodic repetition (McDonald and Simons 1989).<br>• Children able to imitate simple melodic ideas (Marsh and Young 2006).<br>• Songs containing descending intervals and few wide skips are easier to sing (McDonald and Simons 1989).<br>• Attention is focused on melody (Costa-Giomi 2003).<br>• Children can detect mis-tunings in tonal melodies but fail to notice contour-preserving changes as different (Lamont 2009). Contour is not as salient as other musical elements like pitch height and timbre (Creel 2014)	• To provide developmentally appropriate structure to the music experience.<br>• To create expectation through the use of the melodic structure as to initiate a prompt for a lyric-based instruction (e.g. identify an explicit ER strategy) or behavioral expectation (e.g. waiting for a response).<br>• To create expectation through the use of melodic structure that allows an opportunity to practice inhibitory control.	<u>General Description</u><br>• Developmentally appropriate melodies should incorporate melodic repetition and an easy-to-follow contour characterized by descending intervals and step-wise movement.<br><br><u>High Arousal</u><br>• Use unexpected melodic elements to elicit attention, such as ascending intervals, wide skips, an intentional mis-tuning, or a pause before a cadential moment.<br>• Use a pause in the melody to create a sense of expectation.<br><br><u>Low Arousal</u><br>Maintain familiar developmentally appropriate characteristics.

	• Spontaneous singing often includes altered melodic transformations of snatches of known songs (Marsh and Young 2006).<br>• It is challenging to verbally discriminate melodic direction (Costa-Giomi and Descombes 1996).<br>• The best “next note” in a melody is one close in pitch (Lamont 2009).<br>• Musically trained children perform better that untrained at discriminating features of unfamiliar melody (Morrongiello, Roes, and Donnelly 1989).		
Pitch	<u>Music Theory</u><br>• Lower-than-normal pitches are associated with sadness and aggression (Huron 2013).<br>• High arousal is associated with rising and/or sharp micro-intonations and harmonic “noise”; low arousal with flat micro-intonations (Juslin and Timmers 2010).<br><br><u>Music Development</u><br>• Younger children focus on concrete melodic elements such as pitch (Costa-Giomi 2003) and seem especially dependent on pitch height (Creel 2014).<br>• There are variations in reported preschooler pitch ranges, but easiest range may be one octave between a and a’ (McDonald and Simons 1989).<br>• The ability to pitch accurately develops between ages 4 and 6 (Leighton and Lamont 2006).<br>• Young children are able to maintain scale steps within melodic phrases (Krumhansl and Keil 1982; Morrongiello and Roes 1990).<br>• Children are able to discriminate semitone increments (Trehub, Cohen, Thorpe, and Morrongiello 1986) though it is a challenge to verbally discriminate pitch. However, 3-year-olds	• To use pitch changes to reflect desired arousal level and/or modulation between arousal levels.	<u>General Description</u><br>• Incorporate pitches and pitch intervals that help to create singable melodies: an octave pitch range from a to a’; skips with small-integer ratio intervals such as the octave, perfect fifth, perfect fourth.<br>• Expect a developing ability to sing in tune accurately.<br><br><u>High Arousal</u><br>• Incorporate rising pitches, instruments that produce extraneous harmonic “noise,” and sharp changes in pitch tunings<br>• Incorporate sudden and unexpected changes in pitch characteristics.<br><br><u>Low Arousal</u><br>• Use lower-than-normal pitches and no changes in pitch tuning.

	are able to nonverbally express an understanding of pitch-related elements (Costa-Giomi and Descombes 1996).<br>• Preschoolers detect interval changes more easily in the context of small-integer ratios (e.g. octave 2:1, perfect fifth 3:2, perfect fourth 4:3) (Trehub 2003).<br>• The perception of melodic interval is facilitated by musical training (Morrongiello and Roes 1990; Morrongiello et al., 1989; Tsang, Friendly, and Trainor 2011).<br>• Young children are able to use high pitches and pitch variability as cues to identify musical emotions (Stachó et al. 2013)<br>• Pitch is strongly correlated with happiness (Yrtti 2011). Consonant intervals are associated with positive emotions, dissonant with negative emotions (Trainor and Schmidt 2003).<br>• Children as young as 4 manipulate the pitch of familiar songs when asked to present them in happy or sad way (Schubert and McPherson 2006).		
Rhythm	Music Theory<br>• High arousal is associated with no ritardando, sharp duration contrasts, accents on unstable (i.e., rhythmically unstressed) notes and low arousal with inclusion of a final ritardando, accents on stable (i.e., rhythmically stressed) notes, and soft duration contrasts (Juslin and Timmers 2010).<br>• Rhythmic structure can be used to create expectation (Stevens and Byron 2009).<br><br>Music Development<br>• Songs should contain rhythmic repetition (McDonald and Simons 1989).<br>• Spontaneous singing is characterized by control of rhythmic patterns (Welch 2006) and often includes	• To provide developmentally appropriate structure to the musical experience.<br>• To create an expectation through use of rhythmic structure as to facilitate a prompt for a lyric-based instruction (e.g. identify an explicit ER strategy) or behavioral expectation (e.g. waiting for a response).<br>• To create an expectation through use of rhythmic structure that provides an opportunity to practice inhibitory control.<br>• To facilitate motor-based ER	General Description<br>• Developmentally appropriate rhythmic structure should incorporate rhythmic repetition and utilize mostly binary rhythms.<br>• Expect the ability to synchronize to a beat and to control and alter basic rhythmic patterns<br><br>High Arousal<br>• Rhythmic characteristics can include no ritardando, accents on unstable (i.e., rhythmically unstressed) notes, or sudden and sharp rhythmic changes.

	altered rhythmic transformations of snatches of known songs (Marsh and Young 2006).<br>• Children can successful synchronize to a beat (Drake and Bertrand 2003; Drake et al. 2000) and simple rhythmic patterns (Lamont 2009), especially if the patterns are close to a beat (Drake and Gérard 1989).<br>• Children able to reproduce short binary and ternary rhythmic patterns (Lamont 2009; McDonald and Simons 1989), though ternary patterns may be more difficult (Drake and Bertrand 2003).<br>• Younger children focus on concrete melodic elements such as rhythm (Costa-Giomi 2003).<br>• Young children are able to use rhythmic variability and duration contrasts as cues to identify musical emotions (Stachó et al. 2013)<br><br><u>Music and Motor Processing</u><br>• Auditory-motor coactivation is present in infants (Trainor and Zatorre 2009).<br>• Rhythm perception may involve interactions between auditory and cortical motor areas (e.g. motor cortex, supplemental motor area, and premotor cortex) (Trainor and Zatorre 2009) and activation of bilateral, widely-distributed temporo-parietal areas (Stewart et al. 2009). Metric rhythms may engage the frontal lobe and cerebellum (Peretz and Zatorre 2005).<br>• Cortical motor areas are implicated in rhythm perception and production. The cerebellum and basal ganglia are implicated in controlling motor and perceptual timing (Peretz and Zatorre 2005).	strategies.	• Use more complex ternary rhythmic patterns.<br>• Use a pause in rhythmic pattern to create a sense of expectation.<br><br><u>Low Arousal</u><br>• Rhythmic characteristics can include ritardando at end of song, accents on stable (i.e., rhythmically stressed) notes, and little rhythmic change<br>• Maintain familiar developmentally appropriate musical characteristics.
Dynamics	<u>Music Theory</u><br>• Loud music is associated with high arousal and soft music with low arousal (Gabrielsson and Lindström	• To use loudness level changes to reflect the desired arousal level and/or modulate between arousal	<u>General Description</u><br>• Expect an ability to produce and discriminate loud and soft sounds.

	2010).<br>• Dynamics influence valence, e.g. sadness is soft and aggression is loud (Huron 2013). Depending on valence, high arousal can be associated with loud (happiness/anger) or soft (fear) volumes and little (happiness) or large (fear) loudness variability. Low arousal is associated with softness and small sound level variability (Juslin and Timmers 2010).<br><br>Music Development<br>• Young children are able to discriminate loud and soft sounds (McDonald and Simons 1989).<br>• Young children focus on melody unspecific features such as loudness (Lamont 2009).<br>• Young children manipulate dynamics when asked to sing familiar song in happy or sad way (Schubert and McPherson 2006).<br>• Young children are able to use dynamics and dynamic variability as cues to identify musical emotions (Stachó et al. 2013)	levels.<br>• To use loudness level changes to create an expectation that provides an opportunity to practice inhibitory control.<br>To use loudness level to reflect desired valence or emotional tone.	High Arousal<br>• Depending on the intended emotion, incorporate loud sounds (happiness/anger), soft sounds (fear), little loudness variability (happiness) or large loudness variability (fear).<br><br>Low Arousal<br>• Incorporate soft loudness levels and small loudness variability.
Harmony	Music Theory<br>• Modes are commonly associated with valence differences, major modes with a positive valence and minor modes with a negative valence (Gabrielsson and Lindström 2010).<br>Tonal and harmonic structures can be used to create expectation (Stevens and Byron 2009).<br><br>Music Development<br>• Children have a limited understanding of harmony and tonal hierarchy (Trehub 2006) and an imprecise sense of tonality (McDonald and Simons 1989).<br>• The gradual development of sensitivity to key membership and harmony begins to develop between ages 4 and 5 (Corrigall and Trainor 2014;	• To provide developmentally appropriate structure to the musical experience.<br>• Incorporate major and minor modes to reflect simple positive/negative valence associations.<br>• To use simple dissonant and consonant harmonic progressions as to create an expectation that provides an opportunity to practice inhibitory control.<br>• To create an expectation through use of simple harmonic structure that facilitates a prompt for a lyric-	General Description<br>• Developmentally appropriate music will be primarily diatonic and characterized by a simple harmonic structures and use of consonance.<br>• Expect an imprecise sense of tonality.<br>• If incorporating valence, use major modes to reflect positive emotions and minor modes to reflect negative emotions.<br><br>High Arousal<br>• Use harmonic dissonance to create a sense of expectation.

	Costa-Giomi 2003; Dalla Bella, Peretz, Rousseau, and Gosselin 2001; Trehub 2009; Tsang et al. 2011) without conscious awareness (Krumhansl and Keil 1982). Neural evidence indicates sensitivity to tonal structure at age 4 and children acquire sensitivity to key membership before harmony (Corrigall and Trainor 2014).<br>• Sensitivity to mode is established by age 6 and remains unchanged across the lifespan (Dalla Bella et al. 2001).<br>• The acquisition of tonal hierarchy develops in an orderly fashion, starting with the discrimination of scale and nonscale tones (Krumhansl and Keil 1982).<br>• Young children might shift keys between phrases (Krumhansl and Keil 1982; Morrongiello and Roes 1990) but they begin a developmental shift towards being able to maintain key across phrases (Krumhansl and Keil 1982; Morrongiello and Roes 1990; Tsang et al. 2011).<br>• Young children have a predisposition to consonance, but more sensory consonance rather than culture-based harmonic consonance (Costa-Giomi 2003).<br>• Children show a preference for diatonic versus nondiatonic tones (Morrongiello and Roes 1990; Trehub et al. 1986).<br>• Sensitivity to harmony is enhanced by music training (Morrongiello and Roes 1990).<br>• Younger children are unable to distinguish happy and sad music that contrasts by mode (Dalla Bella et al. 2001; Lamont 2009; Schubert and McPherson 2006).	based instruction (e.g. identify an explicit ER strategy) or a behavioral expectation (e.g. waiting for a response).	<u>Low Arousal</u><br>• Maintain familiar developmentally appropriate musical characteristics.
Form	<u>Music Theory</u><br>• Not referenced in literature	• To create expectation through the use of phrasings to support a	<u>General Description</u><br>• Developmentally appropriate music

	Music Development<br>• Children exhibit a developmental shift from incorporating some repetition and ostinato to commonly using rhythmic and melodic repetition (McDonald and Simons 1989).<br>• Children able to group music into segments by pitch, intensity, tone duration, and pause duration (Drake and Bertrand 2003).	prompt for a lyric-based instruction (e.g. identify an explicit ER strategy) or a behavioral expectation (e.g. waiting for a response).<br>• To provide developmentally appropriate structure to the musical experience.<br>• To create an expectation through use of phrasings that provides an opportunity to practice inhibitory control.	characterized by simple structure that includes rhythmic and melodic repetition.<br><br>High Arousal<br>• Use pauses in phrasing to create a sense of expectation.<br><br>Low Arousal<br>Maintain familiar developmentally appropriate musical characteristics.
Tempo	Music Theory<br>• Slow tempos are consistently association with low activation and fast tempos with high activation (Gabrielsson and Lindström 2010; Juslin and Timmers 2010)<br>• Depending on valence, small tempo variations are associated with happiness or anger and large variations with fear (Juslin and Timmers 2010).<br>• Tempo is implicated in the recognition of emotional tone (Peretz and Zatorre 2005).<br><br>Music Development<br>• Children able to detect small tempo changes (Drake and Bertrand 2003) and are responsive to tempo (Marsh and Young 2006).<br>• Young children focus on melody unspecific features such as tempo (Lamont 2009).<br>• Accuracy of synchronized tapping with tempo changes increases with age (Drake, Riess Jones, and Baruch 2000).<br>• Exhibit a developmental shift from being unable to distinguish happy and sad music that contrasts by tempo to being able to do so (Dalla Bella et al.	• To use tempo changes to reflect the desired arousal level and/or modulate between arousal levels.<br>• To incorporate fast and slow tempos to reflect simple positive and negative valence associations.<br>• To provide a temporal structure that facilitates motor-based ER strategies.	General Description<br>• Expect the ability to synchronize motor movements to tempo.<br>• Expect the ability to detect tempo changes and synchronize to them.<br><br>High Arousal<br>• Use fast tempos.<br>• Depending on valence, use small tempo variations (happiness/anger) or large tempo variations (fear).<br><br>Low Arousal<br>• Use slow tempos.

	2001; Lamont 2009).<br>• Children show evidence of an ability to manipulate the tempo of familiar songs when asked to present them in a happy or sad way (Schubert and McPherson 2006).<br>• Children use faster tempos when singing in an angry (Yrtti 2011) or happy way (Mote 2011; Yrtt 2011) and they use tempo as a cue in identifying musical emotions (Mote 2011; Stachó et al. 2013).		
Timbre	<u>Music Theory</u><br>• Tones with higher harmonics are associated with high activation and tones with lower harmonies are associated with low activation (Gabrielsson and Lindström 2010).<br>• High activity is associated with bright or sharp timbres, rising/sharp micro-intonations, and fast/shallow/regular vibrato. Low activity is associated with soft or dull timbres, flat micro-intonations, and slow vibrato (Juslin and Timmers 2010).<br><br><u>Music Development</u><br>• Children are able to discriminate, identify, and remember timbres (Creel 2014; Lamont 2009; McDonald and Simons 1989), though less successfully on difficult comparisons (Lamont 2009).<br>• Children exhibit a rapid development of timbral sensitivity (Lamont 2009).<br>• Young children focus on melody unspecific features such as timbre (Lamont 2009).	• To use timbral qualities to reflect the desired arousal level and/or modulate between arousal levels.<br>• To incorporate timbral changes to facilitate attention-based ER strategies.	<u>General Description</u><br>• Expect a developing ability to discriminate timbres.<br><br><u>High Arousal</u><br>• Use bright/sharp timbres, rising/sharp microintonations, and fast/shallow/regular vibrato.<br>• Incorporate novel timbres or unexpected timbral changes to elicit attention.<br><br><u>Low Arousal</u><br>• Use familiar timbres, soft/dull timbres, and slow vibrato.
Style	<u>Music Theory</u><br>• High activity is associated with staccato articulations (Gabrielsson and Lindström 2010;	• To use articulation style to reflect the desired arousal level and/or modulate between arousal levels.	<u>General Description</u><br>• Developmentally appropriate music can sound like popular music, can

	Juslin and Timmers, 2010), sharp amplitude envelopes (Gabrielsson and Lindström 2010), large articulation variability, and fast or abrupt tone attacks (Juslin and Timmers 2010). Low activity is associated with legato articulations (Gabrielsson and Lindström 2010; Juslin and Timmers 2010), round amplitude envelopes, which are also associated with sadness or softness (Gabrielsson and Lindström 2010), small articulation variability, and slow tone attacks (Juslin and Timmers 2010).<br><br><u>Music Development</u><br>• Children incorporate two broad singing styles, a communicative, chant-like, repetitive style often produced in group activity and a solitary, free-flowing style used primarily in solo and introspective singing (Marsh and Young 2006).<br>• Children are highly accurate at discriminating musical style, especially for popular music (Marshall and Hargreaves 2007).<br>• Young children are able to use tone attacks, vibrato, and articulation a cues to identify musical emotions (Stachó et al. 2013)<br>• Children tend to prefer popular music (Miyamoto 2007).	• To use developmentally appropriate musical style to reflect the desired arousal level and/or modulate between arousal levels.	be chant-like and repetitive, or can be solitary and free-flowing.<br>• Expect an ability to discriminate styles.<br><br><u>High Arousal</u><br>• Use staccato articulations, fast/abrupt attacks, and articulation variability.<br>• Incorporate sudden and unexpected changes to the musical style.<br><br><u>Low Arousal</u><br>• Use legato articulations, slow attacks, small articulation variability.<br>• Use familiar-sounding, developmentally appropriate musical styles.
Lyrics	<u>Music Theory</u><br>• Not applicable<br><br><u>Music Development</u><br>• Children pay more attention to the words rather than the melody when the cognitive load of a task is high (Tsang et al. 2011).<br><br><u>Music and Speech Processing</u><br>There are shared processing areas between those that	• To use lyrics to verbally reflect the intended arousal level.<br>• To use lyrics to verbally prompt the use of explicit ER strategies (attention-based, motor-based, or social assistance-based).<br>• To use lyrics to verbally prompt a exploration of the effectiveness of an ER strategy.	<u>General Description</u><br>• Expect preschoolers to attend to the words.<br>• Use lyrics to verbally prompt the use of explicit ER strategy and/or an exploration of the effectiveness of an ER strategy.<br><br><u>High Arousal</u><br>• The choice of words in lyrics

	underlie speech and language function and music perception and production, namely Broca's area, Wernicke's area, and primary auditory cortex (Devous et al. 2006; Patel 2009; Peretz and Zatorre 2005; Stewart et al. 2009; Trainor and Zatorre 2009).		should reflect high arousal level.<br><br><u>Low Arousal</u><br>The choice of words in lyrics should reflect low arousal level.
Texture	<u>Music Theory</u><br>• Not referenced in literature<br><br><u>Music Development</u><br>• Children are able to recognize texture associated with accompanied versus unaccompanied melodies (McDonald and Simons 1989).	• To incorporate textural changes to facilitate attention-based ER strategies.	<u>General Description</u><br>• Expect a developing ability to discriminate textural changes.<br><br><u>High Arousal</u><br>• Incorporate novel textures or unexpected textural changes to elicit attention.<br>• Use more complex textures, such as an accompanied melody or multiple instruments.<br><br><u>Low Arousal</u><br>• Use simpler textures, such as an unaccompanied melody.

Objective synthesis of the music:

<u>General Description</u>
A developmentally appropriate music stimulus should be predictable and structured, incorporating rhythmic and melodic repetition and simple consonant harmonies. Melodies should have an easy-to-follow contour characterized by descending intervals and step-wise movements. Pitches and pitch intervals should help create "singable melodies" by falling within an octave pitch range and include skips with small-integer ratio intervals (e.g. octaves, perfect fifths, perfect fourths). The music should mostly incorporate binary rhythms, should have a simple form, and should be primarily diatonic. Stylistically appropriate music should sound like popular music, be chant-like and repetitive, or be solitary and free-flowing.

Preschool children can be expected to synchronize to a beat, control and alter basic rhythmic patterns, and produce and discriminate loud and soft sounds. Most will have an imprecise sense of pitch, but will posses a developing ability to sing in tune. Preschool children should be able to synchronize their motor movements to a musical beat, detect tempo changes, and synchronize to those tempo changes. Furthermore, they should be able to discriminate various musical styles, timbres, and textural changes.

If incorporating valence into the music-based experience (e.g. a musically induced positive or negative emotion), major modes can be used to reflect positive emotions and minor modes negative ones. Preschoolers can be expected to focus on words, thus lyrics provide a verbal prompt to use an explicit ER strategy or explore the effectiveness of an ER strategy.

This synthesis outlining developmentally appropriate music is based on the following literature: Costa-Giomi 2003; Costa-Giomi and Descombes 1996; Dalla Bella et al. 2001; Devous et al. 2006; Drake and Bertrand 2003; Drake and Gérard 1989; Drake et al. 2000; Gabrielsson and Lindström 2010; Krumhansl and Keil 1982; Lamont 2009; Leighton and Lamont 2006; Marsh and Young 2006; Marshall and Hargreaves 2007; McDonald and Simons 1989; Miyamoto 2007; Morrongiello and Roes 1990; Morrongiello, Roes, and Donnelly 1989; Patel 2009; Peretz and Zatorre 2005; Schubert and McPherson 2006; Schwarzer 1997; Stewart et al. 2009; Trainor and Schmidt 2003; Trainor and Zatorre 2009; Trehub 2003; Trehub 2006; Trehub 2009; Trehub et al. 1986; Tsang et al. 2011; Welch 2006a.

High Arousal

Highly arousing music avoids ritardandos and accents on unstable notes. For preschool children, it may include complex ternary rhythmic patterns. High arousal music can also have rising pitches and use instruments that produce extraneous harmonic "noise." It typically will be in a fast tempo with bright or sharp timbres, rising or sharp micro-intonations, fast or shallow vibrato, staccato articulations, quick and abrupt attacks, complex musical textures, or with variable articulation styles. Lyrics can be created to reflect the intended arousal level.

To manipulate a music stimulus to make it more arousing, sudden and unexpected musical events or novel musical elements can be incorporated. These can be melodic (e.g. ascending intervals, wide skips, and intentional mis-tunings), pitch-related (e.g. sharp change in pitch tuning), timbral (e.g. novel timbres or unexpected timbral changes), stylistic (e.g. sudden change in musical style), rhythmic (e.g. sudden or sharp rhythmic changes), or textural (e.g. novel texture or unexpected textural changes).

If incorporating valence into the music experience, happy-sounding music can be loud, have small tempo variations, and variability in loudness levels. Angry music can be loud and have small tempo variations. Fearful-sounding music can be soft and have substantial variability in terms of volume or tempo.

This synthesis outlining high arousal music is based on the following literature: Costa-Giomi 2003; Creel 2014; Corrigall and Trainor 2014; Dalla Bella et al. 2001; Devous et al. 2006; Drake and Bertrand 2003; Gabrielsson and Lindström 2010; Huron 2013; Juslin and Timmers 2010; Lamont 2009; Morrongiello and Roes 1990; Morrongiello, Roes, and Donnelly 1989; Mote 2011; Patel 2009; Peretz and Zatorre 2005; Schubert and McPherson 2006; Stachó, Saarikallio, Van Zijl, Huotilainen, and Toiviainen 2013; Stevens and Byron 2009; Stewart et al. 2009; Trainor and Schmidt 2003; Trainor and Zatorre 2009; Trehub et al. 1986; Yrtti 2011.

Low Arousal

Structuring music to create a calming, low arousal effect may be as simple as incorporating the familiar developmentally appropriate musical characteristics previously outlined. Other music characteristics that may be considered low arousal for preschoolers includes music in a lower-

than-normal range, no changes in pitch tuning, soft loudness levels, narrow loudness variability, familiar, soft, or dull timbres, slow tempos, legato articulations, slow attacks, simpler textures, slow vibrato, and limited articulation variability. Rhythmic characteristics of low arousal music include incorporating a ritardando at the end of a song, placing accents on stable notes, and avoiding rhythmic change. Additionally, lyrics can be created to reflect the intended arousal level.

This synthesis outlining low arousal music is based on the following literature: Costa-Giomi and Descombes 1996; Creel 2014; Devous et al. 2006; Drake, Riess Jones and Baruch 2000; Gabrielsson and Lindström 2010; Huron 2013; Juslin and Timmers 2010; Mote 2011; Patel 2009; Peretz and Zatorre 2005; Schubert and McPherson 2006; Stewart et al. 2009; Trainor and Schmidt 2003; Trainor and Zatorre 2009; Yrtti 2011.

References

Costa-Giomi, Eugenia. 2003. "Young Children's Harmonic Perception." *Annals of the New York Academy of Science 999*: 477-484. doi:10.1196/annals.1284.058

Costa-Giomi, Eugenia, and Valerie Descombes. 1996. "Pitch Labels with Single and Multiple Meanings: A Study with French-Speaking Children." *Journal of Research in Music Education 44*(3): 204-214.

Corrigall, Kathleen A., and Laurel J. Trainor. 2014. "Enculturation to Musical Pitch Structure in Young Children: Evidence from Behavioral and Electrophysiological Methods." *Developmental Science 17*(1): 142-158. doi:10.1111/desc.12100

Creel, Sarah C. 2014. Tipping the Scales: Auditory Cue Weighting Changes Over Development." *Journal of Experimental Psychology 40*(3): 1146-1160. doi:10.1037/a0036057

Dalla Bella, Simone, Isabelle Peretz, Luc Rousseau, and Nathalie Gosselin. 2001. "A Developmental Study of the Affective Value of Tempo and Mode in Music." *Cognition, 80*: B1-B10.

Devous, Michael D., Dianne Altuna, Nicholas Furl, William Cooper, Gretchen Gabbert, Wei Tai Ngai, Stephane Chiu, Jack M. Scott, III, Thomas S. Harris, J. Kelly Payne, and Emily A. Tobey. 2006. "Maturation of Speech and Language Functional Neuroanatomy in Pediatric Normal Controls." *Journal of Speech, Language, and Hearing Research 49*(4): 856-866. doi:10.1044/1092-4388(2006/061)

Drake, Carolyn, and Daisy Bertrand. 2003. "The Quest for Universals in Temporal Processing in Music." In *The Cognitive Neuroscience of Music,* edited by Isabelle Peretz and Robert J. Zatorre, 21-31. Oxford: Oxford University Press.

Drake, Carolyn, and Claire Gérard. 1989. "A Psychological Pulse Train: How Young Children Use this Cognitive Framework to Structure Simple Rhythms." *Psychological Research 51*: 16-22.

Drake, Carolyn, Mari Riess Jones, and Clarisse Baruch. 2000. "The Development of Rhythmic Attending in Auditory Sequences: Attunement, Referent Period, Focal Attending." *Cognition 77*: 251-288.

Gabrielsson, Aalf, and Erik Lindström. 2010. "The Role of Structure in the Musical Expression of Emotions." In *Handbook of Music and Emotion: Theory, Research, Applications*, edited by Patrik N. Juslin and John J. Sloboda, 367-400. Oxford: Oxford University Press.

Hanson-Abromeit, Deanna. 2015. "A Conceptual Methodology to Define the Therapeutic Function of Music." *Music Therapy Perspectives*. Available ahead of print. doi: 10.1093/mtp/miu061

Huron, David. 2013. "*Scales and Modes: A Psychological Perspective."* Paper session presented at the meeting for the Society for Music Perception and Cognition, Toronto, Canada, August 8-11.

Juslin, Patrick N., and Renee Timmers. 2010. "Expression and communication of emotion in music performance." In *Handbook of Music and Emotion: Theory, Research, Applications*, edited by Patrik N. Juslin and John A. Sloboda, 453-489. Oxford: Oxford University Press.

Krumhansl, Carol L. and Frank C. Keil. 1982. "Acquisition of the Hierarchy of Tonal Functions in Music." *Memory and Cognition 10*(3): 243-251.

Lamont, Alexandra. 2009. "Music in the School Years." In *The Oxford Handbook of Music Psychology,* edited by Susan Hallam, Ian Cross, and Michael Thaut, 235-243. Oxford: Oxford University Press.

Leighton, Geraldine and Alexandra Lamont. 2006. "Exploring Children's Singing Development: Do Experiences in Early Schooling Help or Hinder?" *Music Education Research 8*(3): 311-330.

Marsh, Kathryn, and Susan Young. 2006. "Musical Play." In *The Child as Musician: A Handbook of Musical Development*, edited by Gary E. McPherson, 289-310. Oxford: Oxford University Press.

Marshall, Nigel A., and David J. Hargreaves. 2007. "Musical Style Discrimination in the Early Years." *Journal of Early Childhood Research 5*(1): 32-46. doi:10.1177/1476718X07072150.

McDonald, Dorothy T., and Gene M. Simons. 1989. *Musical Growth and Development: Birth through Six*. New York, NY: Schirmer Books.

Miyamoto, Karen A. 2007. "Musical Characteristics of Preschool-Age Students: A Review of Literature." *Update: Applications of Research in Music Education 26(1):* 26-40. doi:10.1177/87551233070260010104.

Morrongiello, Barbara A., and Caroline L. Roes. 1990. "Developmental Changes in Children's Perception of Musical Sequences: Effects of Musical Training." *Developmental Psychology 26*(5): 814-820.

Morrongiello, Barbara A., Caroline L. Roes, and Faith Donnelly. 1989. "Children's Perception of Musical Patterns: Effects of Music Instruction." *Music Perception 6*(4): 447-462.

Mote, Jasmine. 2011. "The Effects of Tempo and Familiarity on Children's Affective Interpretation of Music." *Emotion 11*(3): 618-622. doi:10.1037/a0022573.

Patel, Aniruddh D. 2009. "Music and the brain: Three links to language." In *The Oxford Handbook of Music Psychology* edited by Susan Hallam, Ian Cross, and Michael Thaut, 208-216. Oxford: Oxford University Press.

Peretz, Isabelle, and Robert J. Zatorre. 2005. "Brain Organization for Music Processing." *Annual Review of Psychology 56*:89-114. doi:10.1146/annurev.psych.56.091103.070225.Schubert and McPherson 2006;

Schubert, Emery, and Gary E. McPherson. 2006. "The Perception of Emotion in Music." In *The Child as Musician: A Handbook of Musical Development* edited by Gary E. McPherson, 193-212. Oxford: Oxford University Press.

Schwarzer, Gudrun. 1997. "Analytic and Holistic Modes in the Development of Melody." *Psychology of Music 25*: 35-56.

Stachó, László, Suvi Saarikallio, Anemone Van Zijl, Minna Huotilainen, and Petri Toiviainen. 2013. "Perception of Emotional Content in Musical Performances by 3-7-Year-Old Children." *Musicae Sciential 17*(4): 495-512. doi:10.1177/102986497617

Stevens, Catherine, and Tim Byron. 2009. "Universals in Music Processing." In *The Oxford Handbook of Music Psychology* edited by Susan Hallam, Ian Cross, and Michael Thaut, 14-23. Oxford: Oxford University Press.

Stewart, Lauren, Katharina von Kriegstein, Simone Dalla Bella, Jason D. Warren, and Timothy D. Griffiths.2009. “Disorders of Musical Cognition.” In *The Oxford Handbook of Music Psychology* edited by Susan Hallam, Ian Cross, and Michael Thaut, 184-196. Oxford: Oxford University Press.

Trainor, Laurel J., and Louis A. Schmidt. 2003. “Processing Emotions Induced by Music.” In *The Cognitive Neuroscience of Music* edited by Isabelle Peretz and Robert Zatorre, 310-324. Oxford: Oxford University Press.

Trainor, Laurel J. and Robert J. Zatorre. 2009. “The Neurobiological Basis of Musical Expectations.” In *The Oxford Handbook of Music Psychology* edited by Susan Hallam, Ian Cross, and Michael Thaut, 171-183. Oxford: Oxford University Press.

Trehub, Sandra. 2003. “Musical Predispositions in Infancy: An Update.” In *The Cognitive Neuroscience of Music* edited by Isabelle Peretz and Robert Zatorre, 3-20. Oxford: Oxford University Press.

Trehub, Sandra. 2006. “Infants as Musical Connoisseurs.” In *The Child as Musician: A Handbook of Musical Development* edited by Gary E. McPherson, 33-49. Oxford: Oxford University Press.

Trehub, Sandra. 2009. “Music Lessons from Infants.” In *The Oxford Handbook of Music Psychology* edited by Susan Hallam, Ian Cross, and Michael Thaut, 229-234. Oxford: Oxford University Press.

Trehub, Sandra E., Annabel J. Cohen, Leigh A. Thorpe, and Barbara A. Morrongiello. 1986. “Development of the perception of musical relations: Semitone and diatonic structure.” *Journal of Experimental Psychology: Human Perception and Performance 12*(3): 295-301.

Tsang, Christine D., Rayna H. Friendly, and Laurel J. Trainor. 2011. “Singing Development as a Sensorimotor Interaction Problem.” *Psychomusicology: Music, Mind, and Brain 21*(1-2): 31-44. doi:10.1037/h0094002

Welch, Graham F. 2006a. “Singing and Vocal Development.” In *The Child as Musician: A Handbook of Musical Development* edited by Gary E. McPherson, 311-329. Oxford: Oxford University Press.

Yrtti, Antti. 2011. “Structural Factors in Preschool Children’s Emotional Expression in Music.” Master’s thesis, University of Jyväskylä, Jyväskylä, Finland). Retrieved June 2, 2013 from https://jyx.jyu.fi/dspace/handle/123456789/27242
